# Mesenchymal Stem Cell-Derived Extracellular Vesicle: A Promising Alternative Therapy for Osteoporosis

**DOI:** 10.3390/ijms222312750

**Published:** 2021-11-25

**Authors:** Cheng-Hsiu Lu, Yi-An Chen, Chien-Chih Ke, Ren-Shyan Liu

**Affiliations:** 1 Core Laboratory for Phenomics and Diagnostics, Kaohsiung Chang Gung Memorial Hospital, Kaohsiung 833, Taiwan; rocket2350@cgmh.org.tw; 2 Department of Medical Research, Kaohsiung Chang Gung Memorial Hospital, Kaohsiung 833, Taiwan; 3 Molecular and Genetic Imaging Core/Taiwan Mouse Clinic, National Comprehensive Mouse Phenotyping and Drug Testing Center, Taipei 112, Taiwan; yachen0414@gmail.com; 4 Institute of Clinical Medicine, National Yang Ming Chiao Tung University, Taipei 112, Taiwan; 5 Department of Medical Imaging and Radiological Sciences, Kaohsiung Medical University, Kaohsiung 807, Taiwan; 6 Department of Medical Research, Kaohsiung Medical University Hospital, Kaohsiung 807, Taiwan; 7 Drug Development and Value Creation Research Center, Kaohsiung Medical University, Kaohsiung 807, Taiwan; 8 Department of Nuclear Medicine, Cheng Hsin General Hospital, Taipei 112, Taiwan; 9 Department of Biomedical Imaging and Radiological Sciences, National Yang Ming Chiao Tung University, Taipei 112, Taiwan; 10 PET Center, Department of Nuclear Medicine, Taipei Veterans General Hospital, Taipei 112, Taiwan

**Keywords:** osteoporosis, mesenchymal stem cells, extracellular vesicles, EV cargo, engineering EV, osteoporosis medications

## Abstract

Osteoporosis is the chronic metabolic bone disease caused by the disturbance of bone remodeling due to the imbalance of osteogenesis and osteoclastogenesis. A large population suffers from osteoporosis, and most of them are postmenopausal women or older people. To date, bisphosphonates are the main therapeutic agents in the treatment of osteoporosis. However, limited therapeutic effects with diverse side effects caused by bisphosphonates hindered the therapeutic applications and decreased the quality of life. Therefore, an alternative therapy for osteoporosis is still needed. Stem cells, especially mesenchymal stem cells, have been shown as a promising medication for numerous human diseases including many refractory diseases. Recently, researchers found that the extracellular vesicles derived from these stem cells possessed the similar therapeutic potential to that of parental cells. To date, a number of studies demonstrated the therapeutic applications of exogenous MSC-EVs for the treatment of osteoporosis. In this article, we reviewed the basic back ground of EVs, the cargo and therapeutic potential of MSC-EVs, and strategies of engineering of MSC-EVs for osteoporosis treatment.

## 1. Introduction

Osteoporosis, a metabolic skeletal disorder that results from the imbalance between bone formation and bone resorption, generally occurs in postmenopausal women and older people [1]. The process of bone remodeling is participated by mainly osteoclasts and osteoblasts, together with other cells including osteocytes, bone lining cells, monocytes, chondrocytes, hematopoietic stem cells, and mesenchymal stem cells (MSCs) [2,3,4]. Owing to the complicated pathology and uninhibited signaling pathway of osteoporosis, the development of medications is floundering. Nevertheless, various remedies were still developed for the treatment of osteoporosis such as bisphosphonate, selective estrogen receptor modulator (SERM), and calcitonin. Among those agents, bisphosphonates (Alendronate, Risedronate, Zoledronic Acid, Ibandronate) are most widely used in preclinical and clinical as the first-line therapy for osteoporosis. However, the limited therapeutic effects with diverse side effects caused by bisphosphonates hindered the therapeutic applications of osteoporosis [5,6,7,8]. The common adverse effects of bisphosphonates include muscle pain, heartburn, nausea, gastric ulcer, and difficulty swallowing. The medication-related osteonecrosis of the jaw (MRONJ) is rare but it is the most severe side effect [9]. Therefore, researchers have continued searching for an alternative therapy for osteoporosis with better efficacy and fewer side effects. One of the opportunities is cell therapy by mesenchymal stem cells (MSCs). MSCs are multipotent stromal cells able to be isolated from various tissues including cord blood tissue, umbilical cord, placenta, adipose tissue, peripheral blood, bone marrow, dental pulp, amniotic fluid, etc. [10,11,12,13,14]. In addition to the stem cell properties of self-renewal and differentiation, MSCs have also shown great potential of therapeutic effects in numerous human diseases, such as bone and cartilage defects, lung diseases, diabetes mellitus, retinal degeneration, stroke, etc. [15,16,17,18]. Recently, MSC-based treatment was even applied on moderate-severe phases of COVID-19 with promising reports [19]. Given that MSCs possess therapeutic benefits in various human diseases, the underlying therapeutic mechanisms are still being fully elucidated. Currently, the mechanisms of MSC-based therapy can be mainly attributed to immunomodulation, differentiation potential, homing to injured sites, and paracrine effect [20,21,22]. Notably, MSC-based cell therapies are recognized as exerting their therapeutic effect in bone regeneration by paracrine effect rather than their differentiation potential over the past decades [23]. Paracrine effect is a mechanism in which MSCs release a large amount of functional molecules that are taken up by damaged tissues or cells, and subsequently benefits angiogenesis, proliferation, inhibits apoptosis as well as inflammation, and in the case of osteoporosis, promotes the osteogenesis [24]. The conditioned medium (CM) of cultured MSCs were widely used to investigate the paracrine effect because a broad spectrum of beneficial factors could be found in the CM [24,25]. Interestingly, with the increasing evidence being revealed, the fascinating nanoscale “vesicles” isolated from CM were proven to carry paracrine factors and predominate the regulation of paracrine effect in tissue regeneration [26,27].

## 2. MSC-EV with Carried Molecules for Diseases Treatment

Extracellular vesicles (EVs) are lipid bound, nano- to micrometer scaled vesicles secreted by almost all cell types. They can be divided into three subgroups by size, biogenesis, release pathways, encapsulated content and function: exosomes (30–200 nm), microvesicles (MVs, 45–1000 nm), and apoptotic bodies (ABs, 1–4 μm) [28,29].

Based on the guideline of MISEV (minimal information for studies of extracellular vesicles) 2018, “extracellular vesicle (EVs)” is an expert consensus term to describe the vesicles that have the characteristics as follows: (1) The vesicles cannot replicate. (2) The vesicles are naturally released from the cell. (3) The vesicles are encapsulated by lipid bilayers. EVs should be characterized by at least three positive markers (one transmembrane/lipid-bound protein is included) with one negative marker. The markers like tetraspanins families (e.g., CD9, CD63, CD81, and CD82), MVB membrane transport (Alix and TSG101), and heat-shock proteins (Hsp70 and Hsp90) are commonly used as EVs’ markers [30,31]. In particular, MSCs-EVs can reflect the markers from their parental cells by expression of CD29, CD44, CD73, and CD90. This cell-type fingerprint not only provides the targets for characterization, but also indicates that MSC-EVs have a similarly potential to MSCs in the treatment of various disease [32,33]. Among these EVs, exosomes have drawn great attention due to the therapeutic potential and medical application of exosomes from certain cell types, such as MSCs or other stem cells. Exosomes are encapsulated by a lipid bilayer membrane with several types of molecules within the exosomal membrane, including integrins, adhesion molecules, lipid, and certain receptors. Inside the exosomes, various types of molecules are encapsulated, including DNA, messenger RNA, microRNA, non-coding RNA, enzymes, cytokines, as well as many other proteins [14]. All these cargos are the materials involved in the cell–cell communication through exosomes trafficking and uptake by recipient cells. Moreover, different types of cells produce exosomes with different content of cargo, which have different effects. There has been strong evidence that exosomes are the main carrier in charge of transporting most of the secreted factors from cells [15]. Currently, exosomes represent an important mode of intercellular communication by serving as vehicles for transfer between cells of membrane and cytosolic proteins, lipids, and RNA [16].

**Figure 1:**
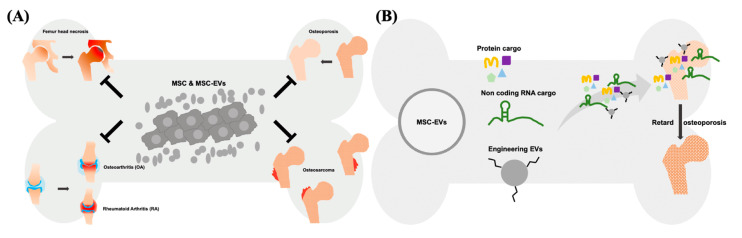
Application of exogenous MSC-EVs on the treatment of bone diseases. (**A**) MSCs and their secreted EVs have been used to successfully treat the femur head necrosis, OA, RA, osteosarcoma, and osteoporosis. (**B**) The retardation of osteoporosis by exogenous MSC-EVs might attribute to carried cargo (protein and non-coding RNA), or exerted by engineering MSC-EVs.

The therapeutic effect of EVs can be exerted via horizontal transfer of molecules, such as proteins, lipids and several types of nucleic acids [34,35,36]. Recently, the therapeutic potential of noncoding RNAs (ncRNAs) has drawn a lot of attention. These ncRNAs include microRNAs (miRNAs), Piwi-interacting RNA (piRNA), long non-coding RNAs (lncRNAs), and others types of secretomes derived from cells. Among these ncRNAs, certain miRNAs and lncRNAs have shown therapeutic potential in numerous diseases [37,38,39]. miRNAs are regulatory small RNAs with 21–23 nt that are involved in posttranscriptional downregulation of protein [40]. They target and inhibit the translation of specific mRNAs and eventually influence the gene expression profile as well as cellular behavior. As the result, EVs’ miRNAs are generally considered the crucial therapeutic cargos in the MSC-EV-based therapy [41,42,43]. The majority of the mammalian genome is transcribed into non-protein-coding mRNAs, including lncRNAs [44]. In the cytoplasm, lncRNAs served as the competing endogenous RNA (ceRNA) to control the binding of miRNA-mRNA, and mediate the translational regulation of mRNA, as well as function as scaffolds of RNA-protein complexes [45,46]. Nuclear lncRNAs mainly influence the organization of chromatin by interacting with related proteins, or prevent the gene loci targeted from specific chromatin factors [47]. As the potential therapeutic targets, several studies demonstrated that lncRNAs play an important role in the treatment of cancer, rare diseases, and infectious diseases [48]. For example, lncRNA H19 carried by MSC-EVs can be transferred to fibroblasts, upregulates the PTEN signaling pathway, and stimulate wound healing in diabetic foot ulcers [49]. The protein cargos of MSC-EVs can be associated with several biological processes of various diseases, particularly with tissue repair and regeneration [50]. EVs derived from WJ-MSCs showed the immunomodulatory capability, mainly through TGF-β and adenosine carried in the EVs which suppress the activation of CD4-expressing T-cells and used for the treatment of canine diseases [51]. Overexpression of hypoxia inducible factor (HIF)-1α in the human dental pulp MSCs promotes the release of EVs, concomitantly carrying overexpressed HIF-1α. These EVs promote angiogenesis by interacting with Notch signaling-rated protein (Jagged1) [52]. Both MSCs and MSC-EVs attenuate ureteral fibrosis by inhibition of TGF-β1/Smad signaling pathway, whereas the therapeutic effect of MSCs might attribute to EVs, the paracrine factors secreted by transplanted MSCs [53]. Of note, MSCs and MSC-EVs act as medications for several bone diseases, such as femur head necrosis, OA, RA, osteosarcoma, and osteoporosis via transferring therapeutic cargo (Figure 1). Collectively, MSC-EVs have shown the curative potential not less than that of parental MSCs in the treatment of numerus disease models, indicating that MSC-EVs is a worth developing medication in the future.

## 3. Applications of Modified EVs for Therapy

In general, the terms “modification” and “engineering” are used to describe the biotechnologies involved in the alteration of the materials for cells, particles, and vesicles [54,55,56]. The common materials include chemical linkers, ligands, proteins, lipids, and nucleic acids. Further, the alteration induced by external pressure such as hypoxia environment, mechanical force, sonochemical reactions, and voltage operations are recognized as the approaches of modification or engineering [57,58,59]. At the genetic level, the term “genetic modification or engineering” can comprehensively cover the process of intentional alteration of genes to produce a beneficial characteristic to a targeted organism [60]. Nevertheless, for the broad spectrum of biomaterials including EVs, nanoparticles (NPs), liposomes, cells, and tissues, there is no uniform description of “modification” and “engineering”. In this review, both “modification” and “engineering” are used to describe the introduction of external materials to EVs or alteration induced by application of pressure. There are a plethora of methods to modify EVs, and loading of exogenous proteins or nucleic acids into EVs are common methods used to explore the feasibility of modified EVs for disease treatment. Physical approaches, including saponin permeabilization, extrusion, sonication, and freeze/thaw cycles, were reported to load hydrophilic molecule (catalase) into EVs [61]. To increase the quantity of EVs, cells cultured in the hypoxic environment secreted EVs with 1.5–2 folds more than that of cells cultured normoxia [62]. Other methods of EV engineering such as surface modification, phospholipid-domain binding, click chemistry, and hybrid EVs with liposomes are introduced to enhance the ability of binding, targeting, and stability of EVs and potentiate the therapeutic effect [63,64,65,66,67]. However, the major challenge of EV engineering is keeping the biological functions of EVs when loading materials or modifying. Several studies showed that loading of exogenous nucleic acids into EV by electroporation might induce the aggregation of EVs and the loaded materials, lower the loading efficiency, and reduce the uptake of EVs by target cells [68,69]. Sonication is an effective method for active loading of hydrophilic agents or nucleic acids into EVs. However, cargo of nucleic acids is easily degraded due to their structural nature and the adverse effect may be resulted by sonication [70,71]. The cargo can be introduced into EVs by incubating with transfection reagent. However, EV membrane might be altered by reagent, which might further affect the delivery of EVs [72,73]. Taken together, suitable modification and engineering of EVs strengthen the therapeutic potential through different aspects, such as enhanced targeting ability or loading of therapeutic agents. Nevertheless, engineering without affecting the bioactivity and function of EVs is always a critical concern.

## 4. Therapeutic Potential of Exogenous MSC-EVs for Osteoporosis

### 4.1. Rebalancing the Bone Homeostasis by Regulation of Bone Formation and Resorption

To date, the strategies of using MSCs or MSC-EVs in the treatment of metabolic bone diseases mainly focus on regulating the bone remodeling by promotion of osteoblasts and inhibition of osteoclasts. Numerous important molecules are involved in the bone remodeling and therefore can serve as the markers for the measurement of dynamic change. These include alkaline phosphatase (ALP), RUNX Family Transcription Factor 2 (RUNX2), collagen, type I, alpha 1 (COL1A1), collagen, type I, alpha 2 (COL1A2), Osteopontin (OPN), osteocalcin (OCN, BGLAP), osterix (Osx, SP7), cathepsin K (CTSK), tartrate-resistant acid phosphatase (TRAP), and calcitonin receptor (CALCR) [74,75,76]. In addition to these markers, there are also signaling pathways regulating the bone remodeling, such as WNT/β-catenin, transforming growth factor-betas (TGF-β), bone morphogenetic proteins (BMP), insulin-like growth factors (IGF), phosphoinositide 3-kinase (PI3K)/Akt, and RANKL/RANK/OPG signaling pathways [77,78,79,80,81]. With the examination of these protein markers or signaling pathways, MSC-EVs are demonstrated to be benefit in the induction of osteogenesis or suppression of osteoclastogenesis. For instance, treatment of EVs derived from human dental pulp stem cells (hDPSCs) promoted the osteogenesis of adipose-derived stem cells (hADSCs) by targeting to MAPK pathway [82]. EVs derived from osteogenic differentiated hADSCs showed enhanced ability to induce osteogenesis of hADSCs. This beneficial loop was verified by upregulated expression of ALP and RUNX2 [83]. In terms of osteoclastogenesis, EVs derived from gingival tissue-derived MSCs (GMSCs) was reported to target to Wnt5a-mediated RANKL pathway, and inhibit the activity and the number of osteoclasts. This therapeutic effect was enhanced after GMSCs are pretreated with tumor necrosis factor alpha (TNF-α) [84]. Despite the promising results from these preclinical studies, so far only few clinical trials of MSC-EVs in the treatment of bone diseases are registered and conducted, as compared with that of MSCs (Table 1). This suggests that the application of MSC-EVs for bone disorders is still at the initial stage, and more evidence regarding the therapeutic effect, targeted signaling pathways, and other mechanisms are needed.

### 4.2. miRNAs and lncRNAs Carried by MSC-EVs Serve as Osteoporosis Medications

For the treatment of osteoporosis, several studies have indicated the therapeutic potential of miRNA in MSC-EVs. A report showed that in the MSC-EVs, 171 miRNAs were discovered which regulated at least 5481 genes and subsequently influenced numerous signaling pathways [85]. After OVX-rats were treated with EVs derived from human bone marrow MSCs (hBMSC-EVs), the study found the increased expression of miR-551b, miR-1263, miR-181b, miR-144, miR-21, and miR-186 in the bone tissues of OVX-rats. Among these, miR-186 was verified to inhibit the expression of MOB Kinase Activator 1A (Mob1) and YAP, which act as the mediators in Hippo signaling pathway [86]. hBMSC-EVs also showed the therapeutic effect with retardation of osteoporosis, by delivery of miR-29b to ovariectomized (OVX) mice. According to the report, MSC-EVs isolated from osteoporotic patients lacked the expression of miR-29b-3p, and therefore the investigators introduced miR-29b-3p into EVs for the treatment of OVX mice. They found that miR-29b-3p-encapsulated EVs targeted and suppressed the nuclear factor kappa B (NF-κB) signaling pathway by inhibiting KDM5A expression, and subsequently improved the osteoporosis in OVX mice [87]. Similar to EVs from bone marrow MSCs, EVs from Wharton’s jelly MSCs have strong potential in the treatment of osteoporosis via miRNA cargo. A study (preprint published on Research Square, DOI: 10.21203/rs.3.rs-37420/v1) proclaimed that WJMSC-EVs’ miR-328-3p and miR-2110 promoted osteogenesis of osteoporotic mice. The report also showed that the PPAR (peroxisome proliferator activated receptor) signaling pathway-associated miR-2110 and let-7c-5p were enriched in WJ-MSC-EVs and regulated osteoclastogenesis [88]. Except for the OVX mice/rats model, osteoporosis treatment by MSC-EVs was also effective in the aged male mice model. EVs secreted from human umbilical cord blood (UCB-EVs) ameliorated bone loss in 16-month-old mice, and it might attribute to the repression of Homeobox A2 by EVs’ miR-3960. Once Homeobox A2, which 1is known to suppress *RUNX2* expression, is downregulated by mir-3960, the promotion of osteoblast differentiation will be activated [89,90]. Another group showed that treatment of mouse BMSCs derived EVs had limited benefit in the increase of bone index of healthy mice, including bone mineral density (BMD), trabecular bone volume (BV/TV), trabecular bone number (Tb.N), and trabecular separation (Tb.Sp) as examined by microCT. However, when loaded with miR-29a, these EVs significantly induced osteogenesis in healthy mice. Further in this study, miR-29a was verified to improve osteoporosis and angiogenesis by directly target to *VASH1* [91]. CBS^+/−^ mice (CBS, Cystathionine β-synthase) tend to behave metabolic bone loss with increased level of homocysteine (Hcy), which is similar to the physical condition of osteoporotic postmenopausal women [92]. When osteoporotic CBS-heterozygous mice were treated with mouse BMSC-EVs, the result revealed that the angiogenesis and osteogenesis of mice were improved via the regulation by lncRNA-H19 (lnc-H19) in BMSC-EVs. Mechanistically, lnc-H19 bound to and inhibited miR-106, which was known to downregulated angiopoietin 1 (*Angpt1*) with the function of bone formation stimulation [93]. Although the achievement of EVs’ miRNAs and lncRNAs based therapy for osteoporosis are impressive, several researches revealed contradictory results and provide another perspective in treatment for diseases by EVs non-coding RNAs cargo. The investigators isolated the EVs from various tissues including plasma, seminal fluid, dendritic cells, mast cells, and ovarian cancer cells for miRNAs quantitative assay, however, most individual EVs did not carry enough amounts of miRNAs to exert their function (According to the result of that research: 0.00825 ± 0.02 miRNA molecules/EV). Needless to say, the individual EV unlikely transfer miRNAs to the cells in their vicinity followed by participating in the down-stream signaling pathway [94]. In addition, a manuscript published on bioRxiv (DOI:10.1101/2020.05.20.106393) also support the concept, based on their quantitative experiments, they did not find strong evidence that miRNA cargo can be delivered by EVs to recipient cells, even engineered EVs could not increase the ability of uptake by cells [95]. Taken together, although the contradictory studies cause the low morale of investigators, it allows investigators to carefully considered their findings, endeavoring in development for EVs’ non-coding RNA based agent for osteoporosis.

### 4.3. MSC-EVs’ Protein Act as Therapeutic Cargo in the Treatment of Osteoporosis

To assess the therapeutic potential of protein carried by MSC-MVs, protein expression profiling performed by proteomic analysis is a useful tool for researchers. The proteomic signatures of MSC-EVs were revealed by extracting EVs from human bone marrow samples at 2012, the study identified 730 EV-carried proteins, and detailed the function into several pathways including MAPK, TGF-β, PPAR, BMP, Wnt, and GF signaling pathways [96]. Interestingly, most of mentioned pathways are related to the pathology of osteoporosis, it elucidated that utilized MSC-EVs protein cargo to develop osteoporosis medications were worthy to pursuit. With increasing studies for proteomic analysis of MSC-EVs, more than 3000 unique proteins have been identified to date. Although the variation of protein cargo in individuals might lead to the difficulties in drug development, the identified cargo still shares similar functional category that provide investigators clear targets to design MSC-EV’ protein-based pharmaceuticals [97,98]. Therefore, a number of researches reported the therapeutic potential of using MSC-EV’ protein cargo in treatment of osteoporosis. OVX mice were used in evaluation of therapeutic effect of human umbilical cord mesenchymal stromal cells-derived extracellular vesicles (hucMSC-EVs) for osteoporosis. A total of 5570 EV-carried proteins were identified by LC-MS/MS analysis, among those cargo, CLEC11A had the highest E/C ratio (EVs/parental cells ratio), allowing it to be a potential target in promotion of osteogenesis. Based on the hypothesis, the group successfully demonstrated that hucMSC-EVs retarded the osteoporosis via transferring CLEC11A. CLEC11A transferred by MSC-EVs not only stimulated progenitor cells to differentiate into osteoblasts, but also participated in regulation of osteoclastogenesis [99]. Moreover, CLEC11A was verified to bind Integrin α11 (Itga11) to stimulate osteogenic activity; however, the investigators did not examine whether CLEC11A carried by exogenous MSC-EVs bind to Itga11 to activate down-stream signaling or not [100]. Moreover, the BMSC-EVs obtained from SD rats were intravenously injected into OVX rats via the caudal vein. Significant improvement of BMD, BV/TV, Tb.N, and Tb.Sp values were revealed after the treatment. The authors further overexpressed the glycoprotein non-melanoma clone B (GPNMB), which facilitated osteoblast differentiation, in the BMSCs and released the EVs with high GPNMB expression (GPNMB-EVs). The result showed that GPNMB-EVs significantly promoted the osteogenesis in vitro and ameliorate the osteoporosis in vivo in comparison with control group (OVX mice without treatment) and BMSC-EVs transfected with empty vector group (NC-EVs) [101]. Given that the BMSC-EVs could attenuate osteoporosis by targeting to Wnt/β-catenin signaling pathway, the authors extended their research in exploration of possible mechanisms of treatment by GPNMB-EVs, and found out that GSK-3β might be the crucial molecule in the treatment. Currently, due to the obstruction of applying MSC in clinical studies, the alternative source of MSC has been explored [102]. With the characteristics of a noninvasive, low-cost, simple procedure, and massive production, the human urine-derived stem cells (USCs) are gradually used in research of stem cells-based therapy [103]. As the result, the therapeutic potential of USCs derived EVs (USC-EVs) for osteoporosis was assessed. Through the transfer of collagen triple-helix repeat containing 1 (CTHRC1) and osteoprotegerin (OPG), USC-EVs effectively promoted the osteogenesis and suppressed the osteoclastogenesis to avoid OVX-induced osteoporotic mice from bone loss [104]. Although knockdown of CTHRC1 or OPG did not totally inhibit the therapeutic effect of USC-EVs due the promiscuous signaling pathway in bone remodeling, the research provided a strong evidence of using EVs’ protein cargo as osteoporosis medications despite USCs are not canonical type of MSC. To date, most studies demonstrated the beneficial effect of MSC-EVs for osteoporosis; however, the contradictory research showed that BMSC-EVs isolated from maxillary bones enhanced osteoclastogenesis [105]. According to their conclusion, the osteoclastogenesis was induced on Raw264.7 cells by treating with BMSC-EVs, whereas the BMSC-EVs were collected from the rats with bone deterioration might not really reflected the function of BMSC-EVs from healthy donors, more comprehensive experiments should be performed to verify the findings. Although non-coding RNAs are widely regarded as most powerful therapeutic candidates in MSC-EV-based medications, investigators should not ignore the value of protein cargo. With the diverse functions and prominent biological activity, protein cargo carried by MSC-EVs are important for investigators to determine the delivery strategy of osteoporosis medications.

## 5. Modification of MSC-EVs in Osteoporosis Therapy

Although MSC-EVs are able to promote angiogenesis, suppress immune response, and support progenitor cell via intracellular delivery of beneficial cargo, other factors such as in vivo stability, targeting ability, and heterogeneous populations might affect the therapeutic effects [106,107,108,109,110]. Nevertheless, studies have shown that the therapeutic efficacy of EVs can be augmented through proper engineering or modifications [111,112]. A number of effective approaches have been applied for the modification of MSC-EVs. For instance, MSC-EVs bound with RGD (Arg-Gly-Asp) peptides hydrogels can boost the effect of acute kidney injury (AKI) repair. After intrarenal injection, the engineered MSC-EVs avoided rapid clearing from circulation in vivo in comparison with non-engineered MSC-EVs, indicating that the RGD modification significantly elevated the stability and prolonged the retention of MSC-EVs [113]. Another group isolated the MSC-EVs from the cells pretreated with lipopolysaccharide (LPS pre-Exo), and examined their therapeutic effect using a cutaneous wound model of diabetic rats. The result showed that LPS pre-Exo enhanced the cutaneous wound healing in diabetic rats. Unique expression of let-7b in LPS pre-Exo induced macrophage polarization by activation of TLR4/NF-κB/STAT3/AKT signaling pathway. Titanium dioxide (TiO2) nanotubes (TNT) are able to promote the cell elongation and differentiation of MSCs. In this report, MSC-EVs were hybridized with TNT (EV-hybrid TNT) for subsequent cell treatment. The result showed that the migratory ability and osteogenic differentiation of hBMSCs were increased by treatment of EV-hybrid TNT. The effect might attribute to the regulation of BMP-2-related signaling pathway due to the finding of upregulated expression of BMP-2 in hBMSCs [114,115]. In another report, Alendronate–extracellular vesicles (Ale-EVs) were synthesized by conjugation of Alendronate–azide group (Ale-N3) and EVs-alkynyl group (EVs-DBCO). Ale-EVs showed the enhanced bone-targeting ability and curative effect for osteoporosis. Meanwhile, MSC-EVs, without Alendronate modification, failed to target to bone tissues of OVX mice. Interestingly, the value of BMD, TV/BV, Tb. Th, Tb. N, and Tb. Sp examined by microCT did not improve by injection of unmodified MSC-EVs in OVX mice, despite that the upregulation of *ALP* and *RUNX2* were noted [116]. Another study showed that conjugation of mouse BMSC-derived EVs with a BMSC-targeting aptamer enhanced the in vivo accumulation in bone tissues. Aptamers are nucleic acids or peptides that target to the specific molecules, and broadly used in basic research, biosensors, molecular imaging and drug delivery [117,118]. MSC-EVs without modification was able to induce in vitro osteoblastic differentiation of BMSCs, while the effect was inefficient to retard the progression of osteoporosis in OVX mice in vivo. However, systemic administration of aptamer-conjugated MSC-EVs, the osteogenic activities of OVX mice were enhanced and osteoporosis was significantly ameliorated. This curative effect might be exerted through the enriched miR-26a carried by MSC-EVs which specifically accumulated in the bone tissue [119]. Together, these results showed the enhancement of curative potential of MSC-EVs after suitable modification. With the increase of studies demonstrates the effective strategies of utilizing naïve MSC-EVs/engineering MSC-EVs cargo for osteoporosis, the downstream signaling mechanisms induced by exogenous MSC-EVs are gradually clarified (Table 2).

## 6. Conclusions and Prospects

In summary, osteoporosis occurs commonly in older populations with an increased risk of fracture and decreased quality of life (QOL). Despite being the first-line medication for osteoporosis treatment, the balance of curative effect and adverse effects of bisphosphonates therapy is not satisfying. MSC therapy is now extensively tested in several diseases in clinical trials, including osteoporosis. MSC-derived EVs offers a strategy of cell-free MSC therapy, exerting the better therapeutic potential in comparison with their parental cells with lower risk of malignant transformation. In this article, we reviewed a number of published references to describe the promising therapeutic effects of MSC-EVs such as promotion osteogenesis and inhibition of osteoclastogenesis in vitro, and retardation of osteoporosis in vivo. Nevertheless, issues such as nomenclature, characterization, the ability scale-up production, and robust quality should be addressed as soon as possible. To accelerate the development of MSC-EV-based medications for osteoporosis, the underlying molecular mechanisms, potential therapeutic targets, timesaving methods, and precise animal models are also needed for better understanding and exploration. Currently, the application of exogenous MSC-EVs in the treatment of osteoporosis is still in the initial stage as compared with that in the treatment of cardiovascular diseases, cancers, lung diseases, kidneys injury, wound healing, and other orthopedic disorders. In recent years, research of cell-free stem cell therapy using MSC-EVs is rapidly advancing, and it may turn into a realistic clinical therapy for osteoporosis in the near future.

## Figures and Tables

**Table 1:** Ongoing clinical trials of MSC and MSC-EVs therapy for bone diseases registered in Clinical Trials.gov. The data were obtained in October 2021.

No.		NCT04501354	NCT04499105	NCT04414592	NCT04759105	NCT05066334	NCT04297813	NCT03692221	NCT04735185	No.		NCT04849429	NCT04998058
Phase	Mesenchymal stem cells (MSCs)	Phase 2	Phase 2	Not Applicable	Phase 2	Phase 2	Phase 3	Early Phase 1	Not Applicable	Phase	Extracellular vesicles (EVs)	Phase 1	Phase 1
Intervention/treatment		Mesenchymal Stem Cell	Mesenchymal Stem Cell + NaCl 0,9% 2 mL	Mesenchymal stem cells	Mesenchymal stem cells	Mesenchymal stem cells	Combination Product: Advanced medicinal Therapy (MSC combined with biomaterial) Procedure: Autologous bone graft	Mesenchymal stem cells	Other: Autologous stem cells Drug: CorticosteroidDrug: Local anesthetic	Intervention/treatment		Biological: Platelet rich plasma (PRP) with exosomes	Procedure: Maxillary sinus floor elevation grafting with synthetic bone substitute.
Intervention model		Single Group Assignment	Single Group Assignment	Single Group Assignment	Parallel Assignment	Parallel Assignment	Parallel Assignment	Parallel Assignment	Parallel Assignment	Intervention Model		Parallel Assignment	Parallel Assignment
Cell sources		Umbilical cord	Umbilical cord	Human umbilical cord	Bone marrow	Bone marrow	-	Bone marrow	Bone marrow	EV sources/term		Platelet-rich Plasma/Exosomes	Adipose tissue-derived mesenchymal stem cells/Conditioned medium
Condition or disease		Osteoporosis	Degenerative Disc DiseaseLow Back Pain Disc Degeneration	Lumbar Disc Degeneration Lumbar Disc Herniation	Intervertebral Disc Degeneration Chronic Low-back Pain	Intervertebral Disc Degeneration Chronic Low-back Pain	Alveolar Bone Atrophy	Disc Degeneration	Chronic Low Back Pain Degenerative Disc Disease	Condition or disease		Chronic Low Back Pain Degenerative Disc Disease	Bone Loss, Osteoclastic Bone Loss, Alveolar Alveolar Bone Loss Alveolar Bone Atrophy Grafting Bone
Last update posted		7 August 2020	6 August 2020	4 June 2020	18 February 2021	4 October 2021	12 March 2020	4 April 2019	10 May 2021	Last Update Posted		19 April 2021	10 August 2021
Sponsor		Indonesia University	Indonesia University	Shanghai General Hospital, Shanghai Jiao Tong University School of Medicine	Campus Bio-Medico University of Rome	Campus Bio-Medico University of Rome	University of Bergen	University Hospitals Cleveland Medical Center	Johns Hopkins University	Sponsor		Dr. Himanshu Bansal Foundation	Pontifical Catholic University of Rio Grande do Sul

**Table 2:** A summary of in vitro and in vivo studies of exogenous MSC-EVs for treating osteoporosis.

Animal Model	Terminology	Source of EVs	Administration Route	EVs Cargos	Duration of Treatment	Time Point of Sacrifice	Target	Refs.
In vitro (steroid-related osteoporosis)	EVs	AFSCs	In vitro model	Not revealed	4 days (Dose not revealed)	Not applicable	Related proteins (SIRT1, FoxO, Nrf2, p21, Galectin-3, AP-1 complex)	[120]
OVX mice	Aptamer-functionalized exosomes	BMSCs	IV injection	miR-26a	100 μg EVs, once per week for 2 months	endpoint of treatment	Not revealed	[119]
Healthy mice	Exosomes	BMSCs	IV injection	miR-29a	100 μg EVs, twice per week for 2 months	endpoint of treatment	*VASH1*	[91]
CBS^+/−^ heterozygous mice	Exosomes	BMSCs	IV injection	lnc-H19	100 μg EVs, 3 times per week for 2 months	endpoint of treatment	miR-106a/*Angpt1*	[93]
OVX rat	Exosomes	BMSCs	IV injection	miR-186	10^13^/mL EVs, once per week for 1 months	endpoint of treatment	Hippo signaling pathway	[86]
In vitro	Exosomes	BMSCs	In vitro model	Not revealed	Not revealed	Not revealed	MAPK signaling pathway	[121]
OVX rat	GPNMB-modified BMSC-EV	BMSCs	IV injection	GPNMB	100 μg EVs, once per week for 2 months	endpoint of treatment	Wnt/β-catenin signaling pathway	[101]
Aged male mice (16 months old)	EVs	hUCB	IV injection	miR-3960	100 μg EVs, once per week for 8 weeks	1, 2 and 8 weeks after the first treatment	HOXA2	[89]
OVX mice	EVs	hucMSCs	IV injection	CLEC11A	100 μg EVs, once per week for 2 months	endpoint of treatment	Integrin α11	[99]
In vitro	EVs isolated from OVX mice with agomiR-miR-29b-3p injection	BMSCs	In vitro model	miR-29b-3p	Not revealed	Not applicable	*KDM5A*/NF-kB pathway	[87]
OVX mice	Exosomes	WJ-MSCs	IP injection	miR-328-3p, miR-2110	0.5 mg/kg EVs, every 3 days for 6 weeks	endpoint of treatment	*CHRD*, *TNF*	[88]
OVX rat	sEV	BMSCs	Not revealed	miR-20a/*BAMBI*	3 weeks (Dose not revealed)	endpoint of treatment	*BAMBI*	[122]

Abbreviation: OVX, ovariectomized; sEV, small extracellular vesicles; AFSC, amniotic fluid stem cells; BMSCs, bone marrow mesenchymal stem cells (BMSCs); hUCB, human umbilical cord blood; hucMSCs, human umbilical cord blood-derived mesenchymal stem cells; WJ-MSCs, Wharton’s jelly mesenchymal stem cells; SIRT1, Sirtuin 1; FoxO, class O of forkhead box transcription factors; Nrf2, nuclear factor erythroid 2-related factor 2; AP-1, activator protein 1; *VASH1*, vasohibin 1; *Angpt1*, angiopoietin 1; MAPK, mitogen-activated protein kinases; GPNMB, glycoprotein non-melanoma clone B; HOXA2, homeobox A2; CLEC11A, C-Type lectin domain containing 11A; *KDM5A*, lysine-specific demethylase 5A; NF-kB, nuclear factor kappa-light-chain-enhancer of activated B cells; *CHRD*, chordin; *TNF*, tumor necrosis factor; *BAMBI*, BMP and activin membrane bound inhibitor.

## Data Availability

Not applicable.
